# The Treatment of Shock by Intravenous Injections

**Published:** 1918-04

**Authors:** 


### SHOCK

---

***The Treatment of Shock by Intravenous Injections.*** By W. M. BAYLISS. Abs. from the *Archives Médicales Belges*, Septembre 1917.

In the search for a substitute for blood to inject in cases of shock, the author believes that one must consider the viscosity and osmotic pressure of colloids. Experiments on animals have shown that Ringer's solution only partially restores the blood pressure because the viscosity is insufficient. If 6 o/o gumarabic is added, however, the pressure is brought up to normal, and, as is not the case with Ringer's solution, it remains constant.

When the osmotic pressure of the colloids of the blood is diminished by dilution in a saline solution, the injected liquid has a tendency to be eliminated through the urine and it also causes edema of the tissues.

For this reason, liquid which is added to give a normal viscosity must also have a definite osmotic pressure. Gum at 7 o/o and gelatine at 6 o/o have the necessary viscosity and also the required osmotic pressure.

Gumarabic is preferable to all other substances because it is easily procurable and can be sterilized. A 2 o/o solution has been used in clinical infusions. Since gumarabic contains no proteins, anaphylactic reactions are very improbable.

In cases of vasodilatation, however, when loss of blood is not involved, injections of gum or of other solutions can be of very little value if used alone. For clinical use a dose of calcium has been added, but the vasoconstricting action of the calcium does not seem as important as that of barium. Pituitrine may also be very useful.

In some cases of shock there is concentration of blood due to the loss of its plasma or of its water or salts only. The injection given must correspond to what has been lost. The general condition of the patient must be considered. It is sometimes well to use a colloid with a lower viscosity and a higher osmotic pressure, such as dextrine.

As the result of the inhalation of certain gases, pulmonary edema may occur. As a result of the liquid thus drawn out of the blood, a concentration and thickening may occur. The liquid found in the lungs in such cases is almost identical with the lymph. To replace this loss in the blood 2 o/o gum solution should be injected intravenously.

In animals, when the blood pressure is lowered by a loss of blood, an aspiration of the tissue liquid takes place, and in this fashion the blood volume is partly restored. This may explain why bleeding in the first stages of gas poisoning gives good results. A certain quantity of liquid can in this manner be removed from the lungs.

In gas gangrene blood cencentration also occurs frequently, indicating a similar procedure.

The author recommends the addition of 9 grammes of sodium chloride per liter of the gum solution, since gumarabic contains chiefly calcium salts and a small amount of potassium salts.

He recommends the use of a " fixe veine " described by Greuze and Grimberg in the *Comptes Rendus de la Société de Biologie*, Feb. 17, 1917. It facilitates the introduction of the needle and prevents it from slipping during the injection. It is to be found at Adnet's in Paris.

